# Heterologous Expression of the *AtNPR1* Gene in Olive and Its Effects on Fungal Tolerance

**DOI:** 10.3389/fpls.2020.00308

**Published:** 2020-03-20

**Authors:** Isabel Narváez, Clara Pliego Prieto, Elena Palomo-Ríos, Louis Fresta, Rafael M. Jiménez-Díaz, Jose L. Trapero-Casas, Carlos Lopez-Herrera, Juan M. Arjona-Lopez, Jose A. Mercado, Fernando Pliego-Alfaro

**Affiliations:** ^1^Instituto de Hortofruticultura Subtropical y Mediterránea “La Mayora”, Departamento de Botánica y Fisiología Vegetal, Universidad de Málaga, Málaga, Spain; ^2^Departamento de Genómica y Biotecnología, Fruticultura Subtropical y Mediterránea (IFAPA), Unidad Asociada de I+D+i al CSIC, Málaga, Spain; ^3^Departamento de Agronomía, College of Agriculture and Forestry (ETSIAM), Universidad de Córdoba, Campus de Excelencia Internacional Agroalimentario ceiA3, Córdoba, Spain; ^4^Instituto de Agricultura Sostenible, Consejo Superior de Investigaciones Científicas, Avenida Menéndez Pidal s/n, Campus de Excelencia Internacional Agroalimentario ceiA3, Córdoba, Spain

**Keywords:** genetic transformation, SAR response, *Olea europaea*, soil-borne pathogens, white root rot, Verticillium wilt

## Abstract

The *NPR1* gene encodes a key component of systemic acquired resistance (SAR) signaling mediated by salicylic acid (SA). Overexpression of *NPR1* confers resistance to biotrophic and hemibiotrophic fungi in several plant species. The *NPR1* gene has also been shown to be involved in the crosstalk between SAR signaling and the jasmonic acid-ethylene (JA/Et) pathway, which is involved in the response to necrotrophic fungi. The aim of this research was to generate transgenic olive plants expressing the *NPR1* gene from *Arabidopsis thaliana* to evaluate their differential response to the hemibiotrophic fungus *Verticillium dahliae* and the necrotroph *Rosellinia necatrix*. Three transgenic lines expressing the *AtNPR1* gene under the control of the constitutive promoter CaMV35S were obtained using an embryogenic line derived from a seed of cv. Picual. After maturation and germination of the transgenic somatic embryos, the plants were micropropagated and acclimated to *ex vitro* conditions. The level of *AtNPR1* expression in the transgenic materials varied greatly among the different lines and was higher in the *NPR1*-780 line. The expression of *AtNPR1* did not alter the growth of transgenic plants either *in vitro* or in the greenhouse. Different levels of transgene expression also did not affect basal endochitinase activity in the leaves, which was similar to that of control plants. Response to the hemibiotrophic pathogen *V. dahliae* varied with pathotype. All plants died by 50 days after inoculation with defoliating (D) pathotype V-138, but the response to non-defoliating (ND) strains differed by race: following inoculation with the V-1242 strain (ND, race 2), symptoms appeared after 44–55 days, with line *NPR1*-780 showing the lowest disease severity index. This line also showed good performance when inoculated with the V-1558 strain (ND, race 1), although the differences from the control were not statistically significant. In response to the necrotroph *R. necatrix*, all the transgenic lines showed a slight delay in disease development, with mean area under the disease progress curve (AUDPC) values 7–15% lower than that of the control.

## Introduction

Olive (*Olea europaea*) is a fruit crop widely cultivated in the countries of the Mediterranean Basin. The species, which is relatively sensitive to cold but highly resistant to heat and drought, was probably domesticated in the Middle East and Central Mediterranean 5500 years B.P. (Rallo et al., 2018). Since then, it has played key roles in the history, economy, culture and environment of this region. Over the last 30 years, the high quality, nutritional properties and health benefits of olive oil (Pérez-Jiménez et al., 2007) have given rise to an increasing interest in this crop linked to changes in its mode of cultivation, such as increasing orchard densities and using selected varieties under irrigation regimes. In addition, olive cultivation has been extended worldwide (Rugini et al., 2011).

Olive culture is threatened by the hemibiotrophic soil-borne fungus *Verticillium dahliae* (Jiménez-Díaz et al., 2012). In a recent estimation with cv. Arbequina in Córdoba, Spain, yield decreases of 84 and 56% were recorded for 3- and 4-year-old affected orchards, respectively (R. M. Jiménez-Díaz and J. L. Trapero-Casas, unpublished data, IAS-CSIC, Córdoba, 2018). Genetic and virulence variation occurring in populations of *V. dahliae* are the main hindrances to the effective management of this disease. Populations of *V. dahliae* comprise two types of pathogenic variation, namely, defoliating (D) and non-defoliating (ND) pathotypes (i.e., symptom types) and pathogenic races 1 and 2 (Jiménez-Díaz et al., 2017). Isolates of the D pathotype cause defoliation of cotton, olive, and okra, whereas isolates of the ND pathotype do not defoliate these species (Jiménez-Díaz et al., 2017). Isolates of race 1, which is avirulent on tomato plants with the resistance gene *Ve1* (de Jonge et al., 2012), belong to the ND pathotype, whereas the Ve1-resistance breaking isolates of race 2 can be found with both D and ND pathotypes (Jiménez-Díaz et al., 2017). According to investigations carried out in tomato (de Jonge et al., 2012), race 1 is characterized by possessing the *Ave1* gene, which encodes a virulence factor that, when it is recognized by the leucine-rich repeat-receptor like protein (LRR-RLP) encoded by the *Ve1* gene, induces the expression of defense mechanisms underlying resistance in the host plant. Race 2 lacks *Ave1* and is pathogenic to cultivars with the *Ve1* gene because a lack of recognition between the factors prevents the induction of defense mechanisms.

The necrotrophic pathogen *R. necatrix*, the causal agent of white root rot, is widely distributed and causes economically important losses in different fruit trees (Sztejnberg, 1980; ten Hoopen and Krauss, 2006); it is also known to affect olive (Sztejnberg, 1980; Guillaumin et al., 1982; García Figueras and Celada Brouard, 2001; Roca et al., 2016). The entire life cycle of *R. necatrix* occurs in the soil, where it can survive for many years. Its mycelia colonize the roots of healthy plants adjacent to infected roots. This fungus invades the plant through the root system, causing generalized rotting of tissues. The symptoms in the aerial parts can develop either quickly or slowly, leading to wilting of leaves, death of branches, and eventually, the death of the tree (Pliego et al., 2012).

Host resistance is the single most practical and efficient method for the management of Verticillium wilt (VW) in olive, but its effectiveness is curtailed by the widespread occurrence of the highly virulent D pathotype in Spain and elsewhere (Jiménez-Díaz et al., 2012). Most widely grown olive cultivars, such as Arbequina and Picual, are highly susceptible to D *V. dahliae*; moreover, the valuable resistance against ND pathotypes found in cultivars such as Frantoio and Changlot Real is overcome by the D pathotype (López-Escudero et al., 2004; Trapero et al., 2015). To the best of our knowledge, no olive selections have been found showing tolerance to *R. necatrix*; for example, García et al. (2009) evaluated 12 different cultivars growing in 2 L containers, and all of them showed a high level of susceptibility to the pathogen.

Plants have different systems of defense against pathogens; among them, SAR (systemic acquired resistance), generated by an increase in SA (salicylic acid) in the site of infection and distal tissues, and ISR (induced systemic resistance), which provides local induced resistance. SAR is established against infections by biotrophic and hemibiotrophic pathogens, with the *NPR1* gene as a key regulator in this pathway (Cao et al., 1997); in Arabidopsis, the NPR1 protein is located in an inactive oligomeric form in the cytoplasm in the absence of pathogens. Following SA-induced changes in the redox state of cells, monomers are released, migrating to the nucleus and inducing SAR signaling, which results in the accumulation of the PR1 (antifungal), PR2 (β-1,3-glucanases), and PR5 (thaumatin-like) proteins (Ali et al., 2018). In contrast, in ISR signaling, jasmonic acid (JA) and ethylene (Et) play key roles. ISR signaling is a defense response against necrotrophic pathogens (Glazebrook, 2005) involving local accumulation of PR3 (Class I, II, IV, V, VI, VII chitinases), PR4 (Class I, II chitinases), and PR12 (defensin) proteins (Ali et al., 2018). However, although SAR and ISR signaling involve different pathways, there exists an overlap between them resulting in either antagonistic or synergistic actions. El Oirdi et al. (2011) found that the necrotrophic fungus *Botrytis cinerea* produces a polysaccharide that, through elicitation of SA signaling and increased *NPR1* expression, inhibits the JA pathway, favoring fungal attack. The suppressive effects of *NPR1* on the JA pathway are well known (Spoel et al., 2003). Alternatively, some toxins imitate JA in silencing the SA pathway, hence increasing the virulence of the pathogen (Zheng et al., 2012). Finally, a synergistic action between the SA and JA signaling pathways has also been demonstrated (van Wees et al., 2000).

The *AtNPR1* gene has been overexpressed in different mono- and dicotyledonous species, inducing resistance to several biotrophic (Cao et al., 1998; Wally et al., 2009), hemibiotrophic (Lin et al., 2004; Makandar et al., 2006; Kumar et al., 2013), and necrotrophic plant pathosystems (Wally et al., 2009; Parkhi et al., 2010b). Taking into account the specificity in the interaction between a species and a given transgene (Islam, 2006), the goal of this investigation was to evaluate the behavior of *AtNPR1* transgenic olive plants following inoculation with either of these two fungi, i.e., the hemibiotrophic *V. dahliae* or the necrotrophic *R. necatrix.*

## Materials and Methods

### Plant Material

Olive embryogenic cultures were established from the radicle of a mature zygotic embryo, cv. Picual, line P1, as indicated by Orinos and Mitrakos (1991). For maintenance, the medium and culture conditions established by Pérez-Barranco et al. (2009) were used, i.e., olive cyclic embryogenesis medium [ECO basal medium containing 1/4 OM (Rugini, 1984) macroelements, 1/4 MS (Murashige and Skoog, 1962) microelements, 1/2 OM vitamins and 550 mg/L glutamine, supplemented with 0.25 μM IBA, 0.5 μM 2iP, 0.44 μM BA and 200 mg/L cefotaxime] in darkness at 25 ± 2°C. Subculturing was carried out at 4-week intervals. The embryogenic culture consisted of small pro-embryogenic masses and globular embryos.

### Binary Vector and Olive Transformation

A DNA fragment containing the *AtNPR1* gene was cloned into the binary plasmid pK7WG2.0, which includes the neomycin phosphotransferase (*nptII)* gene for selection of transformed material. The expression of the *AtNPR1* and *nptII* genes was controlled by the constitutive promoters CaMV35S and NOS (nopaline synthase), respectively. This binary vector was introduced into the disarmed *Agrobacterium tumefaciens* strain AGL1 (Lazo et al., 1991) by the freeze-thaw method (Höfgen and Willmitzer, 1988).

For transformation experiments, *Agrobacterium* cultures were incubated at 28°C in LB medium supplemented with 10 mg/L rifampicin and 100 mg/L spectinomycin at 250 rpm. Prior to somatic embryo inoculation, the bacterial suspension was centrifuged at 4000 × *g* for 10 min, and the pellet was washed with 10 mM MgSO_4_ and finally resuspended in ECO medium at 0.5–0.6 OD_600_.

Genetic transformation experiments were carried out following the protocol described by Torreblanca et al. (2010). A total of 1064 globular somatic embryos (SE) obtained from the embryogenic culture were inoculated with a diluted *Agrobacterium* culture for 20 min under mild agitation. After that, the explants were blotted, dried on sterile filter paper and cultured in ECO solid medium without antibiotics at 25°C in darkness for 48 h. After coculturing, the SEs were washed with ECO liquid medium supplemented with 250 mg/L cefotaxime and timentin at 25°C for 2 h, dried on sterile filter paper and transferred to selection medium, i.e., solid ECO medium supplemented with three antibiotics (250 mg/L cefotaxime, 250 mg/L timentin, and 50 mg/L paromomycin). To select transformed cells, during the first month, embryogenic structures were re-cultured individually onto fresh medium once a week, during the next 2 months at 2-week intervals and monthly afterward. Transformed material was recovered using a progressive selection strategy, i.e., at the beginning, a concentration of 50 mg/L paromomycin was used with progressive increases up to 150 mg/L. Embryogenic lines proliferating in ECO medium supplemented with 150 mg/L paromomycin were grown individually in 25 mL ECO liquid medium supplemented with 250 mg/L cefotaxime and 12.5–25 mg/L paromomycin in an orbital shaker at 120 rpm for 3 weeks. After that, the embryogenic suspensions were filtered through a 2-mm mesh, and the obtained SEs were used for further proliferation in ECO medium. Afterward, 120–150 SE of 1–3 mm in diameter from the selected lines were cultured in maturation ECO medium (basal ECO medium without growth regulators and cefotaxime, supplemented with 1 g/L activated charcoal) over cellulose acetate membranes for 8 weeks as indicated by Cerezo et al. (2011). Then, the mature embryos were transferred to germination medium (Clavero-Ramírez and Pliego-Alfaro, 1990), i.e., modified MS with 1/3 MS macroelements, MS microelements, and 10 g/L sucrose for 12 weeks under 40 μmol⋅m^−2^⋅s^–1^ irradiance level. The obtained shoots were isolated and multiplied in RP medium [DKW macro- and micronutrients as modified by Roussos and Pontikis (2002) and vitamins of Roussos and Pontikis (2002)] supplemented with 2 mg/L zeatin riboside, as indicated by Vidoy-Mercado et al. (2012). Shoots were rooted after a 3-day pulse in basal RP liquid medium supplemented with 10 mg/L IBA and subsequently transferred to basal solid RP medium supplemented with 1 g/L activated charcoal. Acclimatization to *ex vitro* conditions was carried out in jiffy trays with peat moss:perlite (1:1) under a plastic tunnel with gradual lowering of relative humidity for 6–8 weeks. Subsequently, plants were grown in a confined greenhouse with a cooling system, 30°C maximum temperature, and daylight conditions. Plants recovered from non-transformed embryogenic calli were used as controls.

### Phenotypic Analysis of Transgenic Plants

The *in vitro* behavior of the transgenic *AtNPR1* lines was evaluated. For this purpose, 20 shoot segments, 1.5–2 cm in length, with two nodes and deprived of the shoot apex, were isolated from each transgenic line and cultured in RP medium supplemented with 2 mg/L zeatin riboside (Vidoy-Mercado et al., 2012). After 8 weeks, the number of axillary shoots and their lengths were quantified over three subcultures. To evaluate rooting capacity, 2-cm-long apical shoots with at least one node were used. Rooting conditions were as previously described (see section “Binary Vector and Olive Transformation”). After 9 weeks, the number of roots and the length of the main root were measured. The non-transgenic line P1 was used as a control, and the experiment was carried out in triplicate.

Rooted plants were acclimatized to *ex vitro* conditions as previously indicated (see section “Binary Vector and Olive Transformation”). After 9 months of growth in the confined greenhouse under natural daylight conditions, the plant height and the diameter of the main stem were measured. Fourteen plants per transgenic and control line were evaluated.

### Molecular Analysis of Transgenic Plants

The transgenic nature of *AtNPR1* embryogenic lines was confirmed by PCR amplification of a 732-bp fragment from the *AtNPR1* gene and a 700-bp fragment from *nptII*. Genomic DNA was extracted from calli using the protocol of Healey et al. (2014). To amplify the *AtNPR1* gene, the primers used were F: 5′-AATTGAAGATGACGCTGCTCG-3′ and R: 5′-CGACGATGAGAGAGTTTACGG-3′; for *nptII*, the following primers were employed: F: 5′-GAGGCTATTCGGCTATGACTG-3′ and R: 5′-ATCGGGAGCGGCGATACCGTA-3′. All PCRs were prepared in a final volume of 20 μl containing 0.5–1 μl of genomic DNA and 0.5 μM of each primer. Amplification conditions consisted of 4 min at 95°C, followed by 30 cycles of 1 min at 95°C, 45 s at 59°C, and 1 min at 72°C, with a final extension step of 10 min at 72°C.

Approximately 100 mg of *in vitro* leaves from *AtNPR1* transgenic and non-transgenic control lines were collected, powdered in liquid nitrogen and kept at −80°C until used. RNA was extracted using a Spectrum^TM^ Plant Total RNA kit (Sigma-Aldrich) following the manufacturer’s instructions. The concentration and purity of the extracted RNA was assessed using a NanoDrop ND-1000 spectrophotometer (NanoDrop Technologies, Inc., Montchanin, DE, United States). The integrity of RNA samples was visualized on a 1.5% agarose gel under non-denaturing conditions. RNA was treated with DNase I Recombinant, RNase Free (Roche), and cDNA was synthesized by using an iScript^TM^ cDNA Synthesis Kit (Bio-Rad) according to the manufacturer’s instructions.

Quantitative real-time PCR (qRT-PCR) was performed using iTaq^TM^ Universal SYBR Green Supermix (Bio-Rad) in a final reaction volume of 20 μl containing 0.5 μM of each primer and 1 μl of diluted cDNA in a CFX96^TM^ thermocycler (Bio-Rad). For *AtNPR1* amplification, the primers used were designed by Silva et al. (2015) (F: 5′-ATCAGAAGCAACTTTGGAAGGTAGA-3′ and R: 5′-ACCGCCATAGTGGCTTGTTT-3′). The olive ubiquitin gene (F: 5′-ATGCAGATCTTTGTGAAGAC-3′ and R: 5′-ACCACCACGAAGACGGAG-3′) was used as a housekeeping gene for normalization (Gómez-Jiménez et al., 2010). PCR conditions were 30 s at 95°C, 40 cycles of 5 s at 95°C and 30 s at 60°C, followed by a melting curve from 65 to 95°C with 0.5°C increments at 5 s intervals. Relative *AtNPR1* expression levels in *in vitro* leaves were calculated using the 2^–ΔΔCT^ method (Livak and Schmittgen, 2001). An arbitrary value of 1 was given to the line with the lowest gene expression. The experiment was carried out in triplicate, i.e., three independent RNA extractions with three technical replicates for each extraction.

### Western Blot Detection of *AtNpr1*

The *At*NPR1 protein was detected by western blotting in olive leaf extracts from the control and transgenic plants before and after treatment with SA. Twelve-month-old plants growing in a greenhouse were transferred to a growth chamber with a 16 h photoperiod of 150 μmol⋅m^−2^⋅s^–1^ at 25°C 1 week before treatment. Four plants per genotype were sprayed until runoff with a solution of 0.5 mM SA, and leaf samples were taken after 0 and 24 h of treatment. Samples (0.2 g) were extracted in 2 mL extraction buffer (50 mM Tris–HCl, pH 7.5, 150 mM NaCl, 0.5 mM EDTA, 0.1% Triton-X-100, 0.2% Nonidet P-40, 50 μM MG-132 and 50 mM DTT). Homogenates were centrifuged at 23,000 rpm at 4°C for 20 min, and the supernatant was used as the protein extract.

Proteins (60 μg) were separated by SDS-PAGE on 12.5% polyacrylamide gels (Laemmli, 1970), transferred to nitrocellulose membranes (Amersham Protran) using a Trans-blot SD semi-dry transfer cell (Bio-Rad) and blocked with 1% non-fat milk powder at RT. The blots were incubated with a polyclonal antibody against *At*NPR1 (AS12 1854, Agrisera) diluted 1:2500 overnight at 4°C. The membranes were washed three times with blocking solution and incubated with donkey anti-rabbit IgG secondary antibody conjugated with horseradish peroxidase at a dilution of 1:5000. SuperSignal West Femto Maximum Sensitivity Substrate (Thermo Fisher Scientific) was used for chemiluminescence detection. Three independent protein extractions and western blots were performed for the control and each transgenic line.

### Quantification of Endochitinase Activity

Endochitinase activity was measured in young leaves from control and transgenic plants grown in the greenhouse for 10 months. Leaves were powdered in liquid nitrogen and kept at −80°C until use. Samples (0.2 g) were homogenized in 2 mL 50 mM sodium acetate buffer, pH 5.5, with an Ultra-Turrax. Then, the extracts were centrifuged for 20 min at 13,000 rpm and 4°C, and the supernatant was collected. Endochitinase activity was quantified following the protocol of Emani et al. (2003). Protein extracts (100 μL) were incubated with 25 μL of 250 μM fluorescent substrate 4-methylumbelliferyl-β-D-N,N′,N″-triacetylchitotrioside (MUC), dissolved in 0.1 M citrate buffer, pH 3, at 37°C for 1 h in darkness. After incubation, the reaction was stopped with 1 mL of 0.2 M sodium carbonate. Fluorescence was measured in a Clariostar Monochromator Microplate Reader (BMG LABTECH) at 350 nm (excitation) and 450 nm (emission). To quantify endochitinase activity, a standard curve (0.1–10 μM) of the fluorescent compound 4-methylumbelliferone (MU), a product of the hydrolysis of MUC by endochitinases, was carried out. The total protein of each extract was quantified using the Bradford method (Bradford, 1976) with a Bio-Rad Protein Assay following the manufacturer’s instructions. Endochitinase activity was expressed as pmol of 4-MU per hour produced by μg of total protein (pmol 4-MU⋅h^−1^⋅μg of total protein^–1^). Three independent protein extractions per line and three determinations per protein extract were carried out.

### Expression of PR1 Genes in Transgenic Plants

Contigs predicted to encode a basic form of pathogenesis-related protein 1-like in *O. europaea* var. *sylvestris* were searched in the NCBI databases. Those showing homology with regions of PR1-encoding genes from other species were selected for gene expression analysis in leaf tissue from control and transgenic plants by qRT-PCR (Supplementary Table 1).

Primer sequences for the endogenous control genes and the predicted *O. europaea* var. *sylvestris* PR1 genes are shown in Supplementary Table 2. Primer pairs were chosen to generate fragments between 70 and 140 bp and designed using Primer 3 software^1^. Primer specificity was tested by performing conventional PCR and confirmed by the presence of a single melting curve during qRT-PCR. Serial dilutions (1:10, 1:20, 1:50, and 1:200) were made from a pool of cDNA from each treatment and time point, and calibration curves were performed for each gene. For qRT-PCR, the reaction mixture consisted of the first-strand cDNA template, primers (500 nmol final concentration) and SYBR Green Master Mix (SsoAdvanced Universal SYBR Green Supermix, Bio-Rad) in a total volume of 20 μl. The PCR conditions were as follows: 30 s at 95°C, followed by 40 cycles of 15 s at 95°C and 30 s at 60°C, 3 min at 72°C, and 1 min at 95°C. The reactions were performed using an iQ5 real-time PCR detection system (Bio-Rad). The ubiquitin gene was used as an endogenous control for normalization. Relative quantification of the expression levels for the target was analyzed using comparative Ct methods. An arbitrary value of 1 was given to the control, non-transformed line. All reactions were carried out in triplicate.

### *Verticillium dahliae* Infection Assay

Disease reactions to the D and ND *V. dahliae* pathotypes of three *AtNPR1* transgenic lines as well as the non-transgenic control P1, which is highly susceptible to this pathogen (Narvaez et al., 2018), were assessed as previously described (Jiménez-Fernández et al., 2016). Monosporic *V. dahliae* isolates from cotton (V-138: D pathotype, race 2) and olive (V-1242: ND pathotype, race 2; and V-1558: ND pathotype, race 1) (Jiménez-Díaz et al., 2017) were used.

Plants had been grown in 12-cm diameter plastic pots containing a peat moss:perlite substrate (1:1 ratio) and 2 g of Osmocote fertilizer in a confined greenhouse under the natural temperature and photoperiod for over 9 months prior to inoculation with the pathogen. The inoculum consisted of *V. dahliae* mycelia and microsclerotia formed in a cornmeal-sand mixture (CMS; sand:cornmeal:deionized water, 9:1:2, w/w) (Jiménez-Díaz et al., 2017). Inocula were produced in Erlenmeyer flasks containing 400 g of autoclaved (twice at 121°C for 2.5 h) CMS mixture infested with twelve 5-mm-diameter discs from the growing edge of 7-day-old cultures on potato dextrose agar (PDA, Difco Laboratories Inc.) and incubated at 24 ± 1°C in the dark for 1 month. The infested CMS was shaken at 7-day intervals to facilitate the homogeneous colonization of the substrate by the fungus. Then, the infested CMS substrate was thoroughly mixed with a pasteurized soil mixture (clay loam:peat, 2:1, v/v; pH 8.4, 27.5% water holding capacity) at a rate of 10% (v/v) (hereafter referred to as the infested soil mixture). The inoculum density of *V. dahliae* in the infested soil mixture was estimated by serial dilutions on agar plates supplemented with 30 mg/L aureomycin (AAAu) incubated at 24 ± 1°C in the dark for 7 days.

For inoculation, 10-month-old plants were uprooted, and their root systems were washed to remove all the soil. Then, the bare-root plants were transplanted to 13 cm× 13 cm× 12 cm disinfested plastic pots containing the infested soil mixture. Non-inoculated control plants were transferred to a soil mixture containing sterile CMS. After inoculation, the plants were incubated in a growth chamber for variable periods of time, depending on the experiment, at 22 ± 2°C, 60–80% relative humidity and a 14 h photoperiod of fluorescent light of 360 μmol⋅m^−2^⋅s^–1^. The plants were watered every 1–2 days, as needed, and fertilized every 3 weeks with 100 mL Hoagland’s nutrient solution.

Disease reaction in the plants was assessed by the incidence (percentage of plants showing disease symptoms) and severity of foliar symptoms. Symptoms were assessed on individual plants on a 0 to 4 rating scale according to the percentage of affected leaves and twigs (0 = no symptoms, 1 = 1–33%, 2 = 34–66%, 3 = 67–100%, and 4 = dead plant) at 2- to 3-day intervals throughout the duration of the experiments. Disease progress curves and the area under the disease progress curve (AUDPC) were also calculated as described by Campbell and Madden (1990). At the end of experiments, isolations on AAAu were carried out with shoot segments from the inoculated plants to confirm infection of the plant by the pathogen.

Two experiments (I and II) were conducted. Experiment I comprised three *AtNPR1* transgenic lines, the non-transgenic P1 line, and 10-month-old plants of the wild olive clones Ac-15 and Ac-18, previously shown to be highly susceptible and resistant to D *V. dahliae*, respectively. These wild olive lines were used as additional controls to assure adequate experimental conditions for VW development (Jiménez-Fernández et al., 2016). These plants were obtained through micropropagation using the protocol of Vidoy-Mercado et al. (2012). The inoculum consisted of *V. dahliae* isolate 138 (D, race 2), with a mean density in the potting soil mixture of 6.5 × 10^7^ cfu⋅g soil^–1^. The experiment lasted 18 weeks.

A second experiment (Experiment II) was carried out to determine whether the race of the isolate would have an influence on the reaction of transgenic lines to the ND pathotype. Thus, isolates V-1558 (ND, race 1) and V-1242 (ND, race 2) were included for inoculation of the same three *AtNPR1* transgenic lines, together with P1 as a non-transgenic control. The mean inoculum density in the potting soil mixture was 3.1 × 10^7^ cfu⋅g soil^–1^ for isolate V-1558 and 6.0 × 10^7^ cfu⋅g soil^–1^ for isolate V-1242. The experiment lasted 19 weeks.

For each plant genotype, there were 10 and 8 replicated pots (one plant per pot) for inoculated plants in Experiments I and II, respectively, and four non-inoculated plants in each of the experiments, distributed in a completely randomized design.

### *Rosellinia necatrix* Infection Assay

Assays were carried out as described by Ruano Rosa and López Herrera (2009); wheat grains were submerged in distilled water for 24 h and then autoclaved at 121°C and 0.1 MPa for 40 min. Afterward, wheat grains were inoculated with discs from colonies of *Rn* 400 isolate, previously grown on PDA medium, and incubated for 15 days at 24°C in darkness. Over 10-month-old plants growing in a peat moss:perlite (1:1) substrate were inoculated with colonized wheat seeds (1.05 g/L) and grown under greenhouse conditions at 25°C for 2 months. To evaluate the disease reaction, visual symptoms were scored twice a week using a 1–5 scale: (1) healthy plant; (2) leaf chlorosis; (3) first symptoms of wilting and rolling/curling in the leaves; (4) wilted plant with first symptoms of leaf desiccation; and (5) dead plant. The AUDPC values were calculated as described by Campbell and Madden (1990). Ten inoculated plants and 4 non-inoculated plants from each transgenic and control P1 line were used.

### Statistical Analysis

The data were subjected to analysis of variance (ANOVA) using SPSS software version 23. The Levene test for homogeneity of variances was performed prior to ANOVA, and multiple mean comparisons were performed by Tukey’s test. The Kruskal–Wallis test was used for mean comparison in the case of non-homogeneous variances. Pairwise mean comparisons were performed with the Mann–Whitney *U* test. All tests used a significance threshold of *P* = 0.05.

## Results

### Generation of Transgenic *AtNPR1* Olive Plants

**FIGURE 1:**
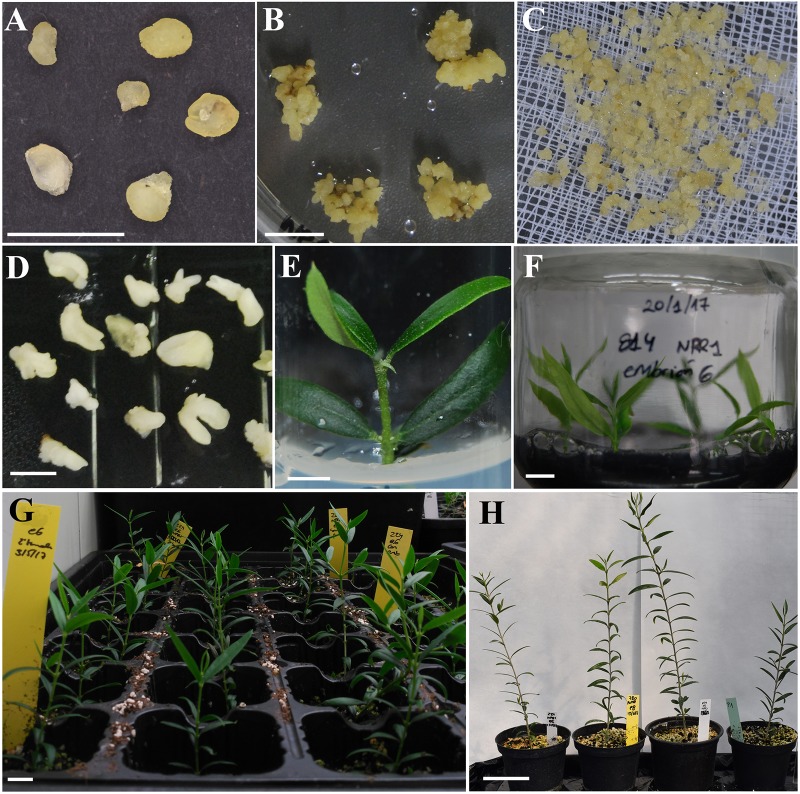
Obtainment of olive lines transformed with the *NPR1* gene from *Arabidopsis thaliana*. **(A)** Globular somatic embryos used for inoculation with *A. tumefaciens*. **(B)** Transgenic callus from the *NPR1*-814 line growing in ECO selection medium supplemented with 150 mg/L paromomycin. **(C)** Transgenic callus from the *NPR1*-814 line after 3 weeks of selection in liquid ECO medium supplemented with 25 mg/L paromomycin. **(D)** Transgenic somatic embryos cultured on maturation medium over a semi-permeable cellulose acetate membrane. **(E)** Micropropagated shoots from the *NPR1*-814 line cultured on RP medium. **(F)** Transgenic *NPR1*-814 shoots cultured on RP medium supplemented with activated charcoal after 3 days in liquid medium with 10 mg/L IBA for rooting. **(G)** Acclimated plants from the *NPR1*-224 line after 6 weeks in the growth chamber. **(H)** From left to right, acclimated plants derived from the transgenic *NPR1*-224, *NPR1*-780, and *NPR1*-814 lines and the non-transformed P1 line after 9 months of growth in the greenhouse. Bars correspond to 0.5 cm **(A,B,D,E)**, 1 cm **(F,G)**, and 5 cm **(H)**.

A total of 1064 globular SEs from the P1 line were inoculated with the *A. tumefaciens* AGL1 disarmed strain carrying the pK7WG2.0 binary vector with the *AtNPR1* gene (Figure 1A). After 24 weeks of culture in solid ECO medium supplemented with 150 mg/L paromomycin, all non-*Agrobacterium*-inoculated embryos were necrotic, while 10 inoculated explants showed proliferation (Figure 1B). These calli were cultured individually in liquid ECO medium supplemented with 12.5–25 mg/L paromomycin for 3 weeks. Then, the calli were filtered, and SEs were transferred to solid ECO medium supplemented with 150 mg/L paromomycin (Figure 1C). Three independent lines proliferated after this additional selection phase, yielding a transformation rate of 0.28%.

After several subcultures, SEs from selected transgenic lines were transferred to ECO basal maturation medium over cellulose acetate membranes (Figure 1D) and germinated in modified MS medium with 1/3 macroelements. The percentages of SE maturation were similar in the control and transgenic lines, approximately 30%, whereas the rates of shoot germination were slightly higher in the transgenic *NPR1* lines, 42–53% vs. 23% in the control. Shoots from all lines could be recovered and micropropagated (Figure 1E) in modified RP medium following the protocol of Vidoy-Mercado et al. (2012). Axillary shoots were rooted (Figure 1F) and acclimatized to greenhouse conditions (Figures 1G,H).

### Molecular Characterization of *AtNPR1* Plants

**FIGURE 2:**
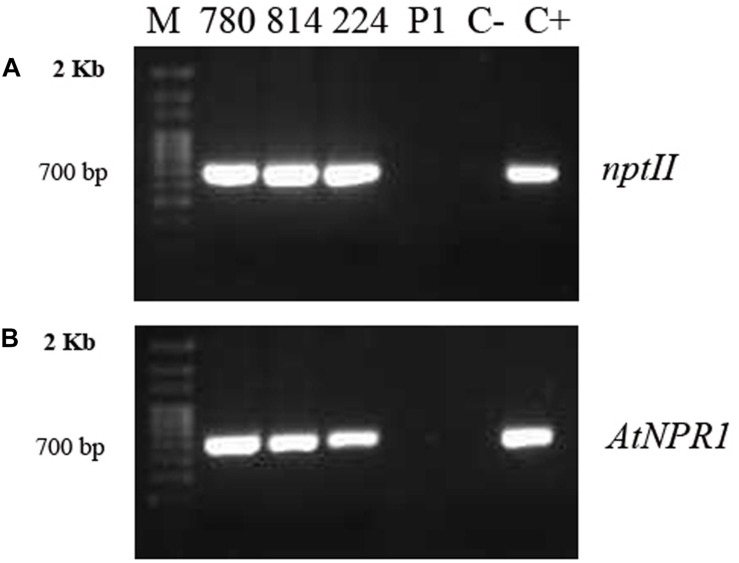
PCR amplifications of the *nptII* **(A)** and *AtNPR1* **(B)** gene fragments from genomic DNA extracted from embryogenic callus from the different transgenic *AtNPR1* lines and non-transformed line P1.780, 814, and 224: transgenic *AtNPR1* lines; P1, non-transgenic control; C–, negative control (without DNA); C+, 35S, *AtNPR1* binary plasmid; M, molecular marker.

Genomic DNA was isolated from embryogenic calli, and PCR amplification of the *nptII* and *AtNPR1* genes was used to confirm their transgenic nature (Figure 2). All transgenic lines amplified a 700-bp fragment from the *nptII* gene (Figure 2A), as well as a 732-bp DNA band corresponding to the amplification of the *AtNPR1* gene (Figure 2B). DNA from the non-transformed control callus did not show any PCR amplification.

**FIGURE 3:**
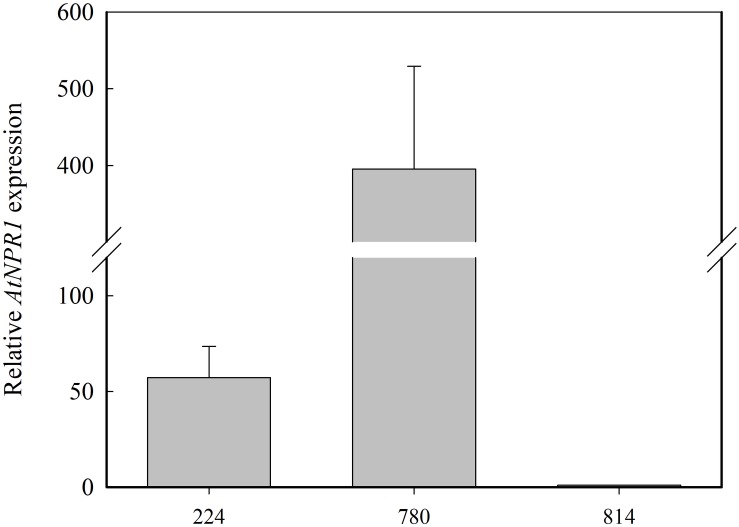
Relative *AtNRP1* expression in leaves from transgenic olive plants. The mRNA values are shown relative to the lowest value obtained in line *NPR1*-814, which was assigned a value of 1. Data correspond to the mean ± SD of three independent RNA extractions.

Expression analysis of *AtNPR1* was carried out with qRT-PCR in RNA extracted from the leaves of the micropropagated transgenic plants (Figure 3). The highest level of *AtNPR1* expression was detected in the *NPR1*-780 line, whereas *NPR1*-224, and *NPR1*-814 showed lower values. As expected, *AtNPR1* expression was not detected in the non-transgenic control.

**FIGURE 4:**
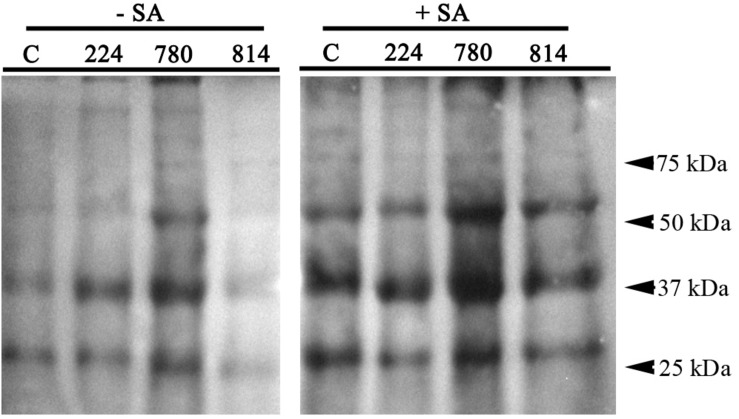
Western blot detection of *At*NPR1 protein in leaf extracts of control (C) and transgenic olive plants. Plants were sprayed with 0.5 mM salicylic acid until runoff, and leaf samples were obtained before (–SA) and 24 h after SA treatment (+SA).

*At*NPR1 was detected in protein extracts from the control and transgenic leaves by western blotting using a commercial *At*NPR1 polyclonal antibody (Figure 4). This antibody recognizes a protein of approximately 66 kDa in *A. thaliana*. A band of this size was detected in the *NPR1*-780 line and, at lower intensity, in *NPR1*-224. Two additional bands of approximately 40 and 28 kDa were also detected in all lines, although their intensity was higher in *NPR1*-780. The NPR1 protein was induced after treatment of the olive plants with 0.5 mM SA for 24 h. The NPR1 band was detected in all the extracts, including the control line (Figure 4).

### PR1 Expression and Endochitinase Activity in Transgenic *AtNPR1* Plants

**FIGURE 5:**
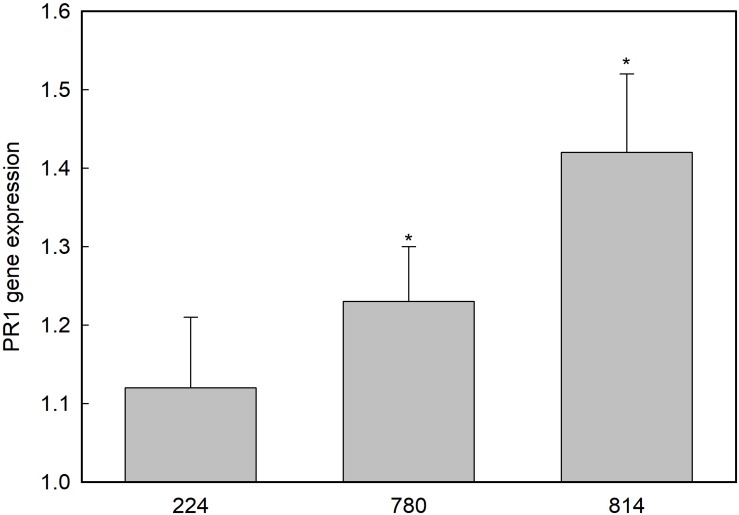
Expression of the PR1-like gene XM_022999257.1 in the leaves of transgenic olive plants. Gene expression is shown as relative to the control non-transgenic plant value, which was given an arbitrary value of 1. Asterisks indicate significant differences from the control by *t*-test at *P* = 0.05.

Among the 11 contigs predicted to encode a basic form of pathogenesis-related protein 1-like in *O. europaea* var. *sylvestris* found in the NCBI database, four of them showed homology to PR1 genes from *Vitis vinifera*, *Prunus mume*, *Medicago truncatula*, and *Jatropha curcas* (Supplementary Table 1). The expression of these genes in leaves was measured by qRT-PCR. Only the gene XM_022999257.1 showed differential expression in control and transgenic plants (Figure 5). This PR1 gene was overexpressed in *NPR1*-780 and *NPR1*-814 lines.

**FIGURE 6:**
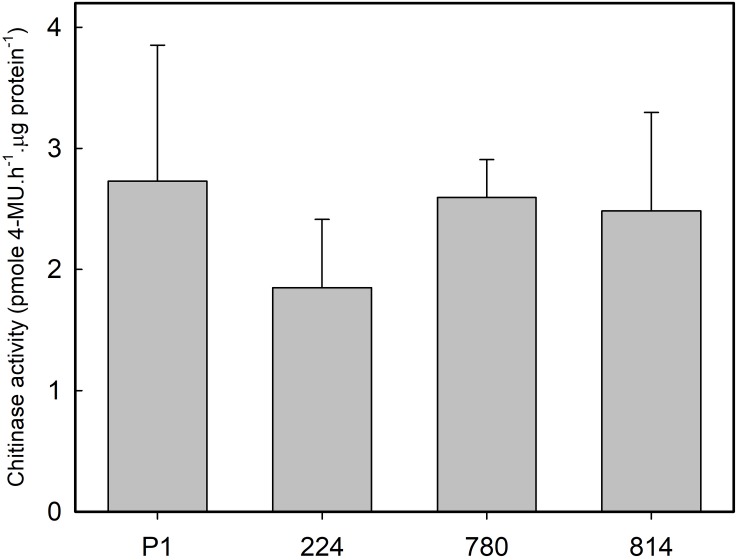
Chitinase activity in the leaves of control (P1) and transgenic olive plants expressing the *AtNPR1* gene. The data correspond to the mean ± SD of three independent protein extractions.

Endochitinase activity was measured in leaves of control and transgenic plants growing in the greenhouse to determine whether the expression of *AtNPR1* was associated with a constitutive ISR response. No significant differences were found in chitinase activity between the control and transgenic lines (Figure 6).

### Phenotypic Characterization of Transgenic *AtNPR1* Plants

To determine the effect of constitutive expression of the *AtNPR1* gene on olive growth, the *in vitro* and *ex vitro* behaviors were examined. For the *in vitro* characterization, axillary shoots were cultured in RP proliferation medium, and several micropropagation parameters were evaluated after 8 weeks of culture (Table 1). Line *NPR1*-814 showed the highest micropropagation rate, as shown by the highest number of shoots per explant and mean shoot length. In contrast, the *NPR1*-780 line showed lower values than the non-transgenic control (Table 1). Rooting was not affected in any transgenic line (Table 1).

**TABLE 1:** *In vitro* characterization of transgenic *AtNPR1* olive plants.

Genotype	Shoots per explant	Length of shoot (cm)	Rooted explants (%)	Roots per explant	Length of main root (cm)
Control	1.9 ± 0.5a	4.2 ± 1.6b	92.1	2.5 ± 1.1a	1.9 ± 1.4
*NPR1*-224	1.9 ± 0.6a	4.2 ± 1.4b	86.2	3.2 ± 1.1a	1.2 ± 0.7
*NPR1*-780	1.6 ± 0.7b	3.9 ± 1.7b	89.5	2.3 ± 1.3a	1.2 ± 0.5
*NPR1*-814	2.1 ± 0.5a	5.1 ± 1.1a	85.4	3.1 ± 1.7a	1.9 ± 1.7

*The data correspond to the mean ± SD. Within each column, means with different letters are significantly different by the Kruskal–Wallis (shoots per explant) or Tukey test (length of shoot) at *P* = 0.05.*

The length and diameter of the main shoot were evaluated in 9-month-old plants growing in the greenhouse. Line *NPR1*-814 showed the highest values for both parameters evaluated, and these values were significantly higher than those of the non-transgenic control (Table 2).

**TABLE 2:** *Ex vitro* characterization of transgenic *AtNPR1* olive plants.

Genotype	Shoot length (cm)	Stem diameter (cm)
Control	18.7 ± 2.6b	0.19 ± 0.02b
*NPR1*-224	18.6 ± 3.6b	0.23 ± 0.02a
*NPR1*-780	19.5 ± 4.2b	0.20 ± 0.02b
*NPR1*-814	23.7 ± 4.3a	0.23 ± 0.01a

*The data correspond to the mean ± SD. Within each column, means with different letters are significantly different by the Tukey test at *P* = 0.05.*

### *Verticillium dahliae* Infection Assay

No symptoms developed in non-inoculated plants in either experiment. In Experiment I, symptoms of VW in the susceptible wild olive Ac-15 started to develop by 3 weeks after inoculation with D *V. dahliae* V-138, and all plants had died 3 weeks later. Similarly, severe disease symptoms developed in control P1 and all *AtNPR1* transgenic lines; these plants started to develop symptoms by 25 days after inoculation, and all plants died 25 days later (results not shown). As expected, the resistant genotype Ac-18 did not develop any disease symptoms.

**FIGURE 7:**
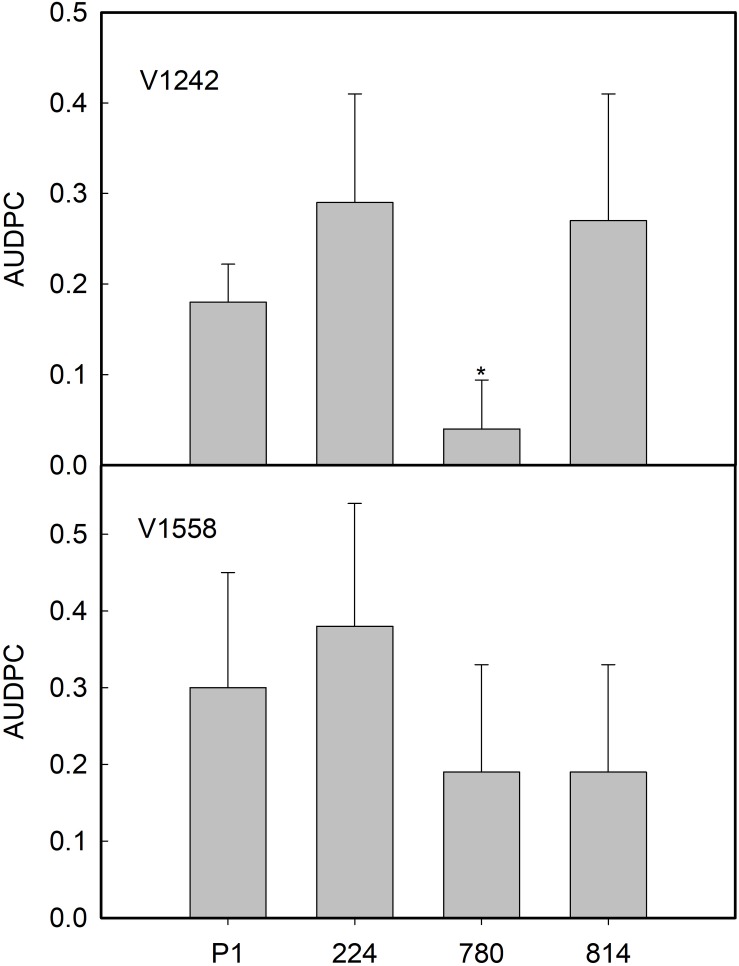
Average values of area under the disease progress curve (AUDPC) in control (P1) and transgenic *AtNPR1* olive plants inoculated with *Verticillium dahliae*, non-defoliating strains V-1242 and V-1558. Data represent the mean ± SE. Asterisks indicate significant differences from control P1 by the Mann–Whitney *U* test at *P* = 0.05.

Subsequently, Experiment II was carried out to determine whether the reaction of the transgenic *AtNPR1* and control P1 lines to the ND pathotypes would be influenced by the race of the isolate. The race 2 ND isolate V-1242 induced the development of first symptoms by 44 and 55 days after inoculation in the *NPR1*-224 and P1 lines, respectively. By 19 weeks after inoculation, the incidence of VW ranged from 50 to 75% in the transgenic lines, while 100% of control plants were affected (Table 3). Interestingly, the severity of symptoms was much lower in *NPR1*-780, the line with the highest relative expression of the transgene, than in the other transgenic lines and the non-transgenic control (Table 3). In addition, the AUDPC mean value in line *NPR1*-780 was lower than those of the other lines (Figure 7). Conversely, the incidence of symptoms induced by race 1 ND isolate V1558 ranged from 50 to 75% in all lines by 19 weeks after inoculation. Following inoculation with this isolate, no significant differences were found among the control P1 and transgenic lines, either in the severity of symptoms (Table 3) or AUDPC values (Figure 7). For both isolates, V-1242 and V-1558, the pathogen could be recovered from virtually all inoculated plants (Table 3).

**TABLE 3:** Response of transgenic *AtNPR1* olive plants to inoculation with *Verticillium dahliae*, non-defoliating strains V1242 and V1558.

*V. dahliae* isolate	Genotype	Plants with symptoms (%)	Symptom severity	Infected plants (%)
V-1242	P1	100a	0.67	100
	*NPR1*-224	75ab	0.90	100
	*NPR1*-780	50b	0.27*	85.7
	*NPR1*-814	62.5ab	0.77	87.5
V-1558	P1	50	0.87	100
	*NPR1*-224	75	1.12	100
	*NPR1*-780	62.5	0.73	87.5
	*NPR1*-814	50	0.43	100

*Asterisks indicate significant differences from the control P1 line by the Mann–Whitney *U* test at *P* = 0.05. The percentages of plants with symptoms were analyzed with the Chi-square test at *P* = 0.05.*

### *Rosellinia necatrix* Infection Assay

**FIGURE 8:**
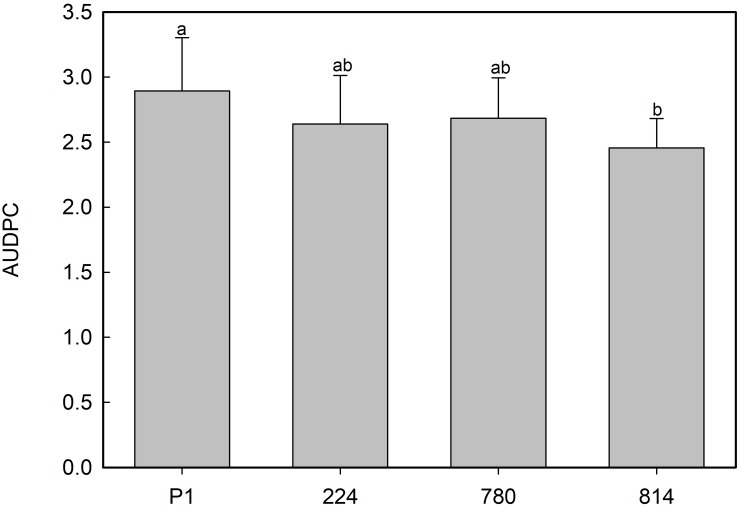
Average values of area under the disease progress curve (AUDPC) in control (P1) and transgenic *AtNPR1* olive plants inoculated with *Rosellinia necatrix*. Data represent the mean ± SE. Different letters indicate significant differences by LSD test at *P* = 0.05.

No symptoms developed in non-inoculated plants. Disease symptoms in plants of non-transgenic control P1 appeared 7 days after inoculation with the *Rn* 400 isolate, and all plants died by 25 days post-inoculation. All transgenic lines showed AUDPC values 7–15% lower than the control, with the best response being observed in the *NPR1*-814 line (Figure 8), although these differences were not significant.

## Discussion

Transformation with genes involved in the regulation of defense pathways against a wide spectrum of pathogens is a useful tool in biotechnological breeding (Lin et al., 2004). In this investigation, olive embryogenic cells were transformed with the *AtNPR1* gene, which is known to play a key role in the SAR response, to evaluate its effect in inducing tolerance to the hemibiotrophic pathogen *V. dahliae* and the necrotrophic fungus *R. necatrix*.

The genetic transformation protocol, including successive exposures to increasing concentrations of paromomycin, was successful in selecting transgenic embryogenic cells. This strategy could be an advantage for selection, avoiding the excessive stress caused in olive cells by *A. tumefaciens* inoculation; this approach favors cell proliferation and results in a better appearance of the callus. The transformation efficiency was 0.28%, whereas in other studies carried out in olive using the same type of explant and the hypervirulent *A. tumefaciens* strain AGL1, transformation rates were higher; for example, Narvaez et al. (2018) obtained a 1.5% transformation rate with the antifungal protein afp of *Aspergillus giganteus*, while with the *FTa1* gene from *M. truncatula* (*MtFTa1*), it was 2.56% (Haberman et al., 2017). These differences in transformation rates could be explained by the size of the transgene. Whereas the sequences of cDNA of *afp* and *MtFTa1* are less than 1 kb, the size of the *AtNPR1* gene, which contains non-coding regions, is approximately 2 kb. Additionally, in the plasmid used, pK7WG2.0, the *nptII* gene is located close to the left border of the T-DNA; therefore, the large size of *AtNPR1* and its location in the right border could hinder the insertion of the *nptII* gene, making selection of transgenic cells more difficult.

The correct expression of the *AtNPR1* gene in transgenic plants was confirmed after detecting *At*NPR1 protein by western blotting, and the protein content in the extracts correlated with gene expression. The amount of *At*NPR1 protein in the transgenic leaves increased after treatment with SA, but unexpectedly, a band of similar size was also detected in the control. This result suggests that *At*NPR1 polyclonal antibody cross-reacts with endogenous olive NPR1; in fact, a search in the *O. europaea* var. *sylvestris* genome yielded two genes (XP_022864634.1 and XP_022864635.1) putatively encoding a BTB/POZ domain and ankyrin repeat-containing NPR1 proteins sharing 53% identity with *At*NPR1. Lin et al. (2004) also used a polyclonal antibody to detect *At*NPR1 in transgenic tomato. In addition to the protein with the expected size, they found in wild-type and transgenic plants that the antibody reacted with other proteins of smaller size, in the range 45 to 35 kDa, which could be derived from endogenous NPR1 homologs.

Overexpression of either *AtNPR1* or its homologs has caused negative effects on the growth or development of plants, i.e., in strawberry, Silva et al. (2015) obtained shorter plants, with reduced canopy size, following transformation with this gene; moreover, the level of transgene expression was negatively correlated with plant size. In olive, the *NPR1*-780 line, with the highest transgene expression and amount of NPR1 protein, showed a lower number of shoots of shorter length under *in vitro* proliferation; however, these differences were not observed in 9 months old plants growing in the greenhouse. Differential behavior under growth chamber or greenhouse conditions was also observed in rice plants overexpressing the *AtNPR1* gene; these plants showed the LMS phenotype (*lesion mimic spot*) when growing in a chamber under low light, while only a growth reduction was noticed following transfer to the greenhouse; moreover, these transgenic rice plants were more sensitive to abiotic stress, such as salinity and drought, as well as to viral infections (Quilis et al., 2008). Molla et al. (2016) reported that some negative effects of *AtNPR1* expression could be avoided by using a specific green tissue promoter instead of a constitutive promoter.

Silva et al. (2015) hypothesized that the reduction in growth and development observed in *AtNPR1*-transformed strawberries could be due to constitutive activation of defense pathways. These transgenic plants constitutively expressed a *PR5* gene (thaumatin); however, their SA content was similar to that of the control plants. Other *PR* genes have also been shown to be constitutively expressed following transformation with this gene or its homologs, i.e., *PR1b* and *PBZ1/PR10* in rice (Chern et al., 2005), *PR1* and genes coding for glucanases (*PR2*) and chitinases (*PR3*) in tomato (Lin et al., 2004). In contrast, constitutive overexpression of *AtNPR1* in Arabidopsis (Cao et al., 1998), wheat (Makandar et al., 2006), or cotton (Kumar et al., 2013) did not induce the expression of *PR* genes in the absence of pathogens or elicitors. In this research, four genes putatively encoding PR1 were identified; only one of them was slightly upregulated in transgenic plants, although the level of expression did not correlate with *AtNPR1* gene expression or protein content. On the other hand, Parkhi et al. (2010a) only found increases in chitinase activity in *AtNPR1* cotton plants following inoculation with *Fusarium oxysporum* or after elicitation with SA. Basal endochitinase activity in leaves was similar in control and transgenic olive plants; hence, *AtNPR1* expression apparently did not induce constitutive expression of *PR* genes coding for these enzymes, at least in the absence of fungal stimulus. According to Niggeweg et al. (2000), variations in the interaction between *NPR1* and bZIP transcription factors (*TGA*) could explain differences in *PR* gene expression.

Regarding the response to *V. dahliae*, a hemibiotrophic fungal pathogen, olive plants expressing the *AtNPR1* gene did not show any resistance to the D isolate (V138), confirming previous observations by Parkhi et al. (2010a) in cotton; however, behavior against ND pathotypes varied with the race of the isolate, i.e., the *NPR1-*780 line with the highest transgene expression showed the lowest AUDPC and severity symptom values following inoculation with ND isolate V-1242 (race 2, absence of the *Ave1* gene). The performance of transgenic plants following inoculation with ND V-1558 (race 1, presence of the *Ave1* gene) was somewhat different, and no significant differences among the control and transgenic lines could be observed. In any case, it appears that the *Ve1* gene is not present in the P1 line, since control plants lack resistance to this pathotype. Moreover, the observed differences between pathotypes could confirm that an alternative infection mechanism depending on the fungal race is operating, as shown by Fradin et al. (2009) in *V. dahliae*-susceptible tomato. Our results in olive are in accordance with the previous observations of Parkhi et al. (2010a) in cotton, where a positive response to ND *V. dahliae* was observed in *AtNPR1* transgenic plants. Resistance to this pathogen seems to involve JA signaling (Fradin et al., 2011; Gao et al., 2013), and overexpression of GhFMO1 (a defense protein in SA signaling) in tobacco increased susceptibility to this fungus (Xu et al., 2014). However, other investigations have shown the importance of SAR signaling in response to *Verticillium*. In potato, overexpression of a polymorphic sequence of cDNA-AFLP, *StoNPR1*, isolated from a *Verticillium*-resistant genotype of *Solanum torvum* (Wang et al., 2010), conferred resistance through induction of genes involved in the SA biosynthetic pathway, including *ICS1* (*isochorismate synthase 1*) (Deng-Wei et al., 2014). Similarly, in cotton, Gong et al. (2017) reported that decreased resistance was observed following silencing of SA-upregulated ribosomal protein gene *L18* (*Ga*RPL18), while Tang et al. (2019) found that silencing of three *Wall Are Thin* genes resulted in increased SA levels and lignin synthesis and, subsequently, enhanced resistance to *V. dahliae*. Our results indicate a beneficial effect of SAR response in one of the olive-ND *V. dahliae* isolates used.

Transformation with *AtNPR1* has successfully been used to induce tolerance to necrotrophic fungi such as *B. cinerea*, *Alternaria radicina*, and *Sclerotinia sclerotiorum* in carrot (Wally et al., 2009) and *Alternaria alternata*, *Rhizoctonia solani*, and *F. oxysporum* in cotton (Parkhi et al., 2010b; Joshi et al., 2017). Defense against necrotrophic pathogens would be dependent on the JA/Et pathways; however, Berrocal-Lobo and Molina (2004) demonstrated that resistance to *F. oxysporum* was mediated by Et, JA, and SA in Arabidopsis and that the *NPR1* gene was involved. Along this line, Mur et al. (2006) indicated that SA-JA interactions in pathogen attack would depend on the relative concentration of the hormones, i.e., transient synergistic effects would be found at low hormonal concentrations, whereas higher concentrations and longer exposure times would result in antagonistic effects.

In Arabidopsis plants overexpressing the *NPR1* gene and inoculated with the necrotrophic pathogen *Fusarium graminearum*, Makandar et al. (2010) found that defense mediated by SA-*NPR1* would contribute to the control of disease, while JA-mediated signaling would favor infection by restricting the activation of defense regulated by SA-*NPR1.* In any case, a positive role of JA in defense could not be rejected since double mutants (*npr1* and insensitive to JA, *jar1*) were more susceptible to the disease than single *npr1* mutants. The fact that this fungus has a short biotrophic phase in the early stages of infection (Goswami and Kistler, 2004) could partially explain the role of SA signaling in defense. In this investigation, one of the transgenic lines showed slower disease progression than the control following inoculation with the necrotrophic fungus *R. necatrix*, suggesting that, in this pathosystem, overexpression of *NPR1* slightly improves the response of plants to the fungus, although no correlation could be found between this positive effect and transgene expression or basal endochitinase activity.

## Conclusion

Heterologous expression of the *NPR1* gene from *A. thaliana* in olive does not confer resistance to a D pathotype of the hemibiotroph fungus *V. dahliae*, although it improved the plant response to ND pathotypes. Similarly, a slight improvement in plant behavior was observed following inoculation with the necrotrophic fungus *R. necatrix*. However, the level of resistance attained in both cases does not make it a feasible approach to control these diseases.

## Data Availability Statement

The datasets generated for this study are available on request to the corresponding author.

## Author Contributions

IN and LF were responsible for obtaining, maintaining, and characterizing the transgenic plants. IN, CP, and EP-R carried out the molecular analysis. RJ-D and JT-C performed the *V. dahliae* assays. CL-H and JA-L carried out the *R. necatrix* assays. JM and FP-A planned this research, designed the experiments, and wrote the manuscript.

## Conflict of Interest

The authors declare that the research was conducted in the absence of any commercial or financial relationships that could be construed as a potential conflict of interest.

## Footnotes

1 http://bioinfo.ut.ee/primer3-0.4.0/
