# Supplementary material for: Heterologous Expression of the AtNPR1 Gene in Olive and Its Effects on Fungal Tolerance
Source: Front Plant Sci. 2020 Mar 20;11:308. doi: 10.3389/fpls.2020.00308 (PMC7100536; doi:10.3389/fpls.2020.00308)
Supplement: Supplementary file 1 [file Data_Sheet_1.PDF]

Heterologous expression of AtNPR1 gene in olive and its effects in fungal tolerance

Narvaez, I.[1], Pliego, C.[2], Palomo-Ríos E.[1], Fresta, L.[1], Jiménez-Díaz, R.M.[3,4], Trapero-Casas, J.L.[4], López-Herrera, C.[4], Arjona-López, J.M.[4], Mercado, J.A.[1], Pliego-Alfaro, F.[1*]

Supplementary Table 1. List of contigs predicted to encode a basic form of pathogenesis-related protein 1-like in *Olea europaea* var. *sylvestris* that showed homology with regions of PR1 encoding genes from other species.

**Accession Number**		**Cover query**	**E-value**	**Per. Ident**
XM_022999257.1	*Vitis vinifera* pathogenesis-related protein PR-1 XM_002276732.4	45%	3e-51	77.62%
XM_023043750.1	*Prunus mume* basic form of pathogenesis-related protein 1-like. XM_008237928.1	10%	7e-09	78.99%
XM_023042687.1	*Medicago truncatula* pathogenesis-related protein 1 XM_013607709.2	17%	2e-07	76.09%
XM_023002290.1	PREDICTED: *Jatropha curcas* pathogenesis-related protein PR-1. XM_012209902.2	52%	2e-87	79.91%

Supplementary Table 2. List of primers used for qRT-PCR quantification of PR1 encoding genes in olive

Primer	Sequence
XM_022999257.1-Forward	5´AAGCAATTCTTGGCTTCACAA 3´
XM_022999257.1-Reverse	5´TAACGGGTTAACCTGGCATC 3´
XM_023043750.1-Forward	5´GCACAGAACTACGCGAATCA 3´
XM_023043750.1-Reverse	5´CTCCCCTTAGCCAGGTTTTC 3´
XM_023042687.1-Forward	5´AACGAAAGGTATGCGGATTG 3´
XM_023042687.1-Reverse	5´AGCAAGATTTCTCCCCAACC 3´
XM_023002290.1-Forward	5´GTGGGCAGGACAGAGAAGAG 3´
XM_023002290.1-Reverse	5´TCCCCAAGCTTGAAGTCATC 3´
Ubiquitin-Forward	5´ATGCAGATCTTTGTGAAGAC 3´
Ubiquitin-Reverse	5´ACCACCACGAAGACGGAG 3´
